# Identification of SMCHD1 domains for nuclear localization, homo-dimerization, and protein cleavage

**DOI:** 10.1186/s13395-018-0172-z

**Published:** 2018-08-02

**Authors:** Yosuke Hiramuki, Stephen J. Tapscott

**Affiliations:** Human Biology Division, Fred Hutchinson Cancer Research Center, Seattle, WA 98109 USA

**Keywords:** Facioscapulohumeral muscular dystrophy, SMCHD1, Nuclear localization, Homo-dimerization, Protein cleavage, DUX4

## Abstract

**Background:**

SMCHD1 is a disease modifier and a causative gene for facioscapulohumeral muscular dystrophy (FSHD) type 1 and type 2, respectively. A large variety of different mutations in SMCHD1 have been identified as causing FSHD2. In many cases, it is unclear how these mutations disrupt the normal function of SMCHD1.

**Methods:**

We made and analyzed lenti-viral vectors that express Flag-tagged full-length or different mutant SMCHD1 proteins to better understand the functional domains of SMCHD1 in muscle cells.

**Results:**

We identified regions necessary for nuclear localization, dimerization, and cleavage sites. Moreover, we confirmed that some mutants increased DUX4 expression in FSHD1 myoblasts.

**Conclusions:**

These findings provide an additional basis for understanding the molecular consequences of SMCHD1 mutations.

**Electronic supplementary material:**

The online version of this article (10.1186/s13395-018-0172-z) contains supplementary material, which is available to authorized users.

## Background

Facioscapulohumeral muscular dystrophy (FSHD) is characterized by weakness initially of the facial, scapular, and upper arm muscles, but progresses to involve most of the skeletal muscles of the body. DUX4 is normally not expressed in the skeletal muscle, whereas it is mis-expressed in the FSHD skeletal muscle [1]. DUX4 is a retrogene encoding a double-homeobox transcription factor and is present in each copy of the D4Z4 macrosatellite repeat, a 3.3 kilobase unit in multicopy arrays in the subtelomeric regions of chromosomes 4 and 10. The most common form of FSHD is caused by a shortened D4Z4 array with ten or fewer units on a permissive haplotype of chromosome 4 (FSHD1) [2, 3]. A phenotypically identical form of FSHD2 is caused by mutations in SMCHD1, a member of the condensin/cohesin family of chromatin factors required for silencing some repetitive regions [4]. FSHD2 also requires the presence of a permissive haplotype of chromosome 4, which is characterized by specific single-nucleotide polymorphisms that create a polyadenylation signal for the DUX4 mRNA [3]. In addition, SMCHD1 is also a disease modifier of FSHD1 [5].

The human SMCHD1 gene consists of 48 exons, and the protein has an ATPase domain in the amino-terminus and a hinge domain in the carboxy-terminus [6]. The ATPase domain hydrolyzes ATP and the hinge domain mediates SMCHD1 dimerization [7–11]. SMCHD1 is a chromatin binding protein that has a role in the epigenetic silencing of the D4Z4 region, the X chromosome, and other regions of DNA repeats in the genome [4, 10, 12–16]. Although multiple different FSHD2-causing mutations in SMCHD1 have been reported, limited knowledge of the functional regions of the SMCHD1 protein restrict our understanding of the consequences of each mutation. Here, to better understand the function of SMCHD1 as a chromatin binding protein, we focused on the identification of the region(s) necessary for nuclear localization and homo-dimerization.

## Methods

### Cell culture

Control (MB135) and FSHD1 (MB073) myoblasts were cultured in growth medium (F-10 including 10% fetal bovine serum, 10 ng/ml human recombinant FGF, 1 μM dexamethasone, 100 U/ml penicillin, and 100 μg/ml streptomycin) or differentiation medium (DMEM including 1% horse serum, 10 μg/ml insulin, 10 μg/ml transferrin, 100 U/ml penicillin, and 100 μg/ml streptomycin). These cells were transduced with the lentivirus of interest using 8 μg/ml polybrene. For RT-qPCR, these cells were selected using 2 μg/ml puromycin. For making lentivirus, 293T cells cultured in DMEM including 10% fetal bovine serum, 100 U/ml penicillin, and 100 μg/ml streptomycin were transfected with a lenti-viral construct vector, psPAX2 (Addgene plasmid no. 12260), and pMD2.G (Addgene plasmid no. 12259) using lipofectamine 3000 following manufacture protocol. For human SMCHD1 cDNA lenti-viral constructs, cDNA for full length or different mutant SMCHD1 purified by PCR or digested by restriction enzymes was inserted into the GFP site of pLenti CMV GFP Puro (Addgene plasmid no. 17448) [17]. Lenti-viral construct that removed GFP from pLenti CMV GFP Puro was used as “No insert” for RT-qPCR. Sequences of the primers are listed in Additional file 1.

### Western blot

For preparation of sample, cells were lysed with lysis buffer (50 mM Tris-HCl pH 6.8, 150 mM NaCl, 0.2% Triton X-100) including protease inhibitors (Roche). Lysates were on ice for 15 min and spun down at 12000 rpm at 4 °C for 15 min. The supernatant was transferred to a new tube, added with 2 × Laemmli sample buffer, and boiled for 5 min. For immunoprecipitation (IP), lysates were pre-cleaned with protein A and protein G mixture (Millipore) at 4 °C for 1 h, immunoprecipitated with anti-Flag antibody (Sigma F1804, 1:300) and protein A and protein G mixture at 4 °C overnight, washed with lysis buffer five times, added with 2 × Laemmli sample buffer, and boiled for 5 min. The sample was resolved on 4–12% Bis-Tris gel (Invitrogen) and transferred to PVDF membrane (Novex), and then, the membrane was exposed to blocking buffer (5% dry milk) for 1 h followed by primary antibody (SMCHD1 (Sigma, HPA039441, 1:3500 or Abcam, ab179456, 1:3500), Flag (Sigma, F1804, 1:3500), HA (Abcam, ab18181, 1:3500), and α-Tubulin (Sigma, T9026, 1:14000)) at 4 °C overnight, and secondary antibody (Jackson ImmunoResearch) at room temperature for 1 h. Signals were detected using ECL Western Blotting Substrate (Thermo Scientific) or West Femto Maximum Sensitivity Substrate (Thermo Scientific) in X-Ray Film Processors (AFP Imaging).

### Immunofluorescence

Cells were fixed with 4% PFA for 12 min, permeabilized with 0.5% Triton X-100 for 13 min, blocked with blocking buffer (0.1% goat serum, 0.1% BSA, 0.02% Tween20), and incubated with primary antibodies (Flag (Sigma, F1804, 1:500), SMCHD1 (Sigma, HPA039441, 1:500), MF20 (DSHB, MF 20 was deposited to the DSHB by Fischman, D.A., 1:500), and H3K27me3 (active motif, 61017, 1:500)) at 4 °C overnight and incubated with secondary antibodies (Jackson ImmunoResearch) at room temperature for 1 h. DAPI (Sigma) was used for staining nucleic acid. Fluorescence was obtained with a Zeiss Axiophot (AxioCam MRm camera and Axiovision 4.6 software).

### RT-qPCR

Total RNA was isolated using NucleoSpin RNA (Macherey-Nagel). After DNase I treatment, cDNA synthesis with oligo dT primers was performed using SuperScript III Reverse Transcriptase (Invitrogen). After RNase H treatment, RT-qPCR was performed using iTaq Universal SYBR Green Supermix (Bio-Rad) on QuantStudio 7 Flex (Applied Biosystems). Sequences of the primers are listed in Additional file 2.

### Statistical analysis

Statistical analysis was carried out using Dunnett’ s test. A value of *P* < 0.01 was considered statistically significant.

## Results

### Identification of the SMCHD1 nuclear localization signal

**Fig. 1:**
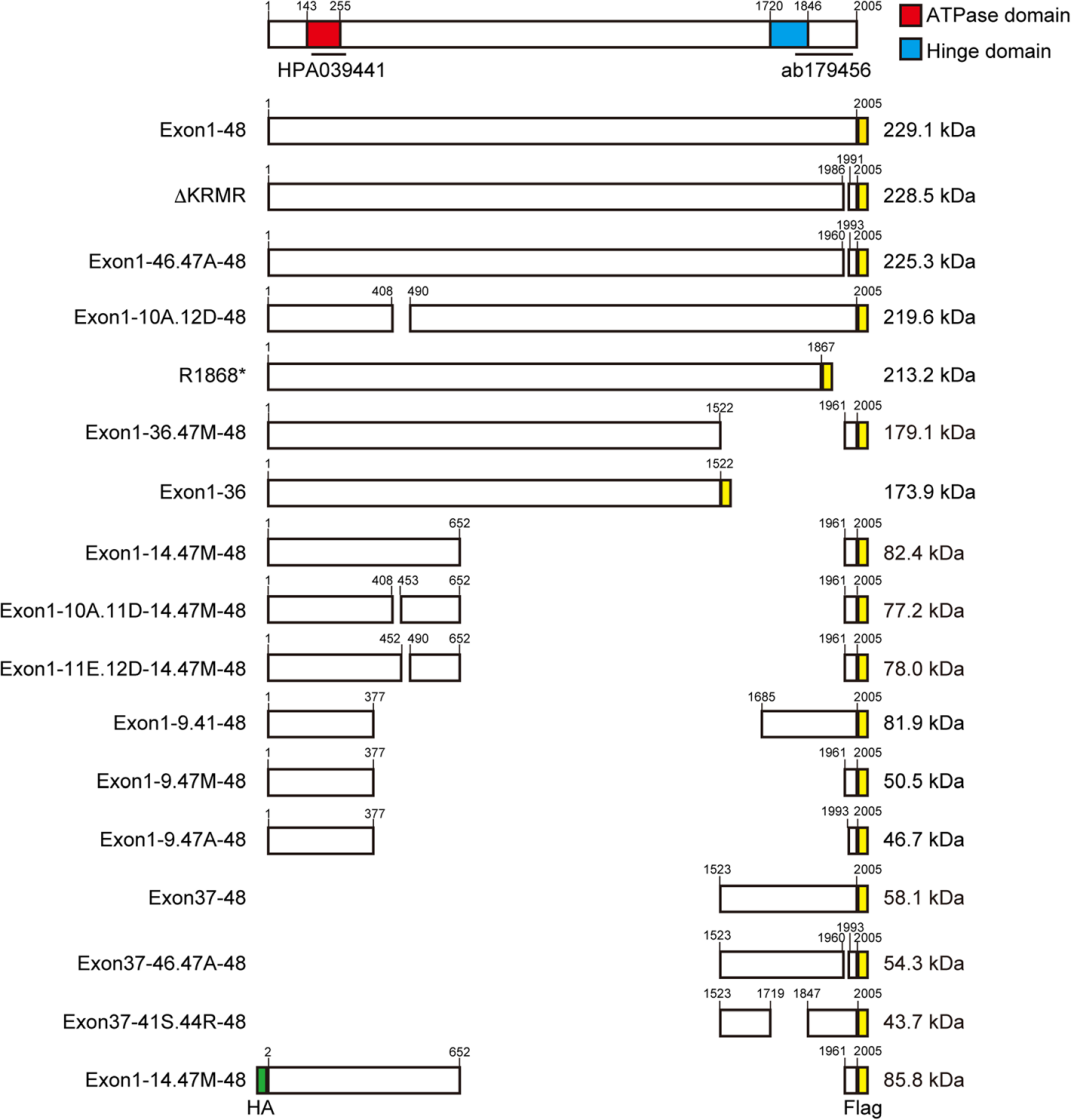
Schematic of full-length and different mutant SMCHD1 constructs. The gene and protein SMCHD1 consists of 48 exons and 2005 amino acids, respectively, and the ATPase domain in the amino-terminus and the hinge domain in the carboxy-terminus are indicated in red and blue, respectively. A bar indicates recognition regions for anti-SMCHD1 antibody (HPA039441 and ab179456). Predicted molecular weight is shown in the right side. 3 × HA tag (green) and 3 × Flag tag (yellow)

**Fig. 2:**
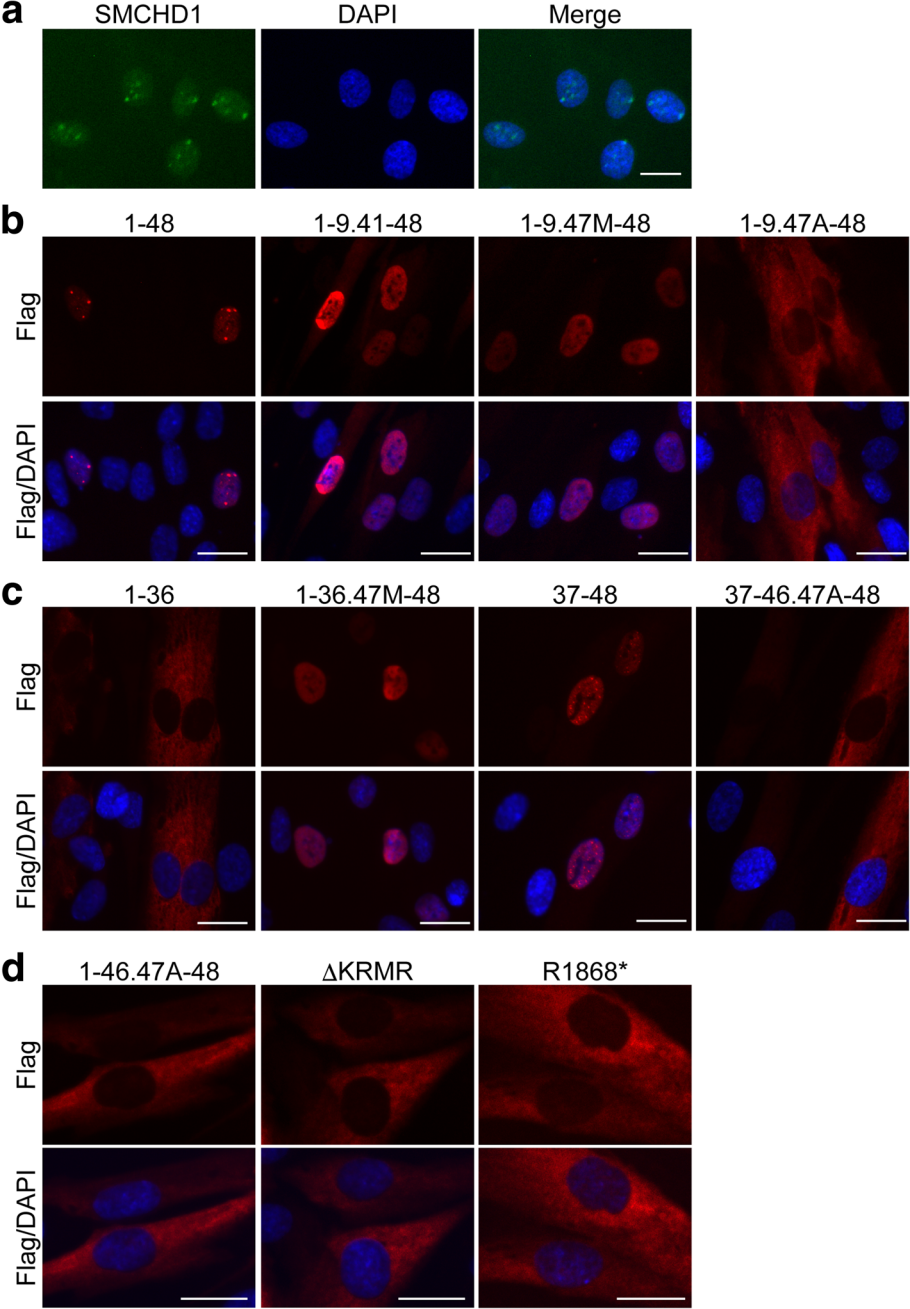
Identification of the nuclear localization signal. **a** Immunofluorescence for SMCHD1 in control myoblasts. **b**–**d** Immunofluorescence for Flag in control myoblasts transduced with Flag-tagged SMCHD1 lentivirus. DAPI was used for staining the nucleus. Scale bar is 20 μm. The regions necessary for nuclear localization were confirmed by repeat experiments

In order to characterize SMCHD1 proteins in muscle cells, we made lenti-viral vectors that express full length or different mutant SMCHD1 protein (Fig. 1). SMCHD1 localized to the nucleus in control (MB135) myoblasts under growth medium (Fig. 2a). To identify the nuclear localization signal (NLS) in SMCHD1, we used immunofluorescence to determine the localization of Flag-tagged full-length and mutant SMCHD1 proteins in control myoblasts. The mutant proteins from constructs Exon1-48-Flag, Exon1-9.41-48-Flag, and Exon1-9.47M-48-Flag were mostly localized to the nucleus, whereas proteins from construct Exon1-9.47A-48-Flag were localized to the cytoplasm (Fig. 2b). Therefore, the 32 amino acids (aa) between residues 1961 and 1992 (MTPIRKCNDSLRHSPKVETTDCPVPP**KRMR**RE) functioned as an NLS. This NLS was sufficient for SMCHD1 to localize to the nucleus because Exon1-36-Flag was expressed in the cytoplasm and Exon1-36.47M-48-Flag, which has NLS sequence, was expressed mostly in the nucleus (Fig. 2c). Similarly, Exon37-48-Flag was expressed mostly in the nucleus, while Exon37-46.47A-48-Flag, which lacks the NLS, was expressed in the cytoplasm (Fig. 2c). Moreover, we deleted this NLS from full-length SMCHD1 (Exon1-46.47A-48-Flag) and confirmed that it was expressed in the cytoplasm (Fig. 2d), which suggests that this NLS is necessary for SMCHD1 to localize to the nucleus. As these 32 amino acids include the consensus sequence of K(K/R)X(K/R) for a classical NLS (bold in the sequence above) [18], we deleted these four amino acids (KRMR) from full-length SMCHD1 (∆KRMR) and confirmed that it was expressed in the cytoplasm (Fig. 2d). A FSHD2 causing mutation of SMCHD1 introduces a stop codon between the hinge domain and the nuclear localization signal (R1868*) [19] and expression of a tagged version of this mutation (R1868*-Flag) showed mostly cytoplasmic localization (Fig. 2d).

**Fig. 3:**
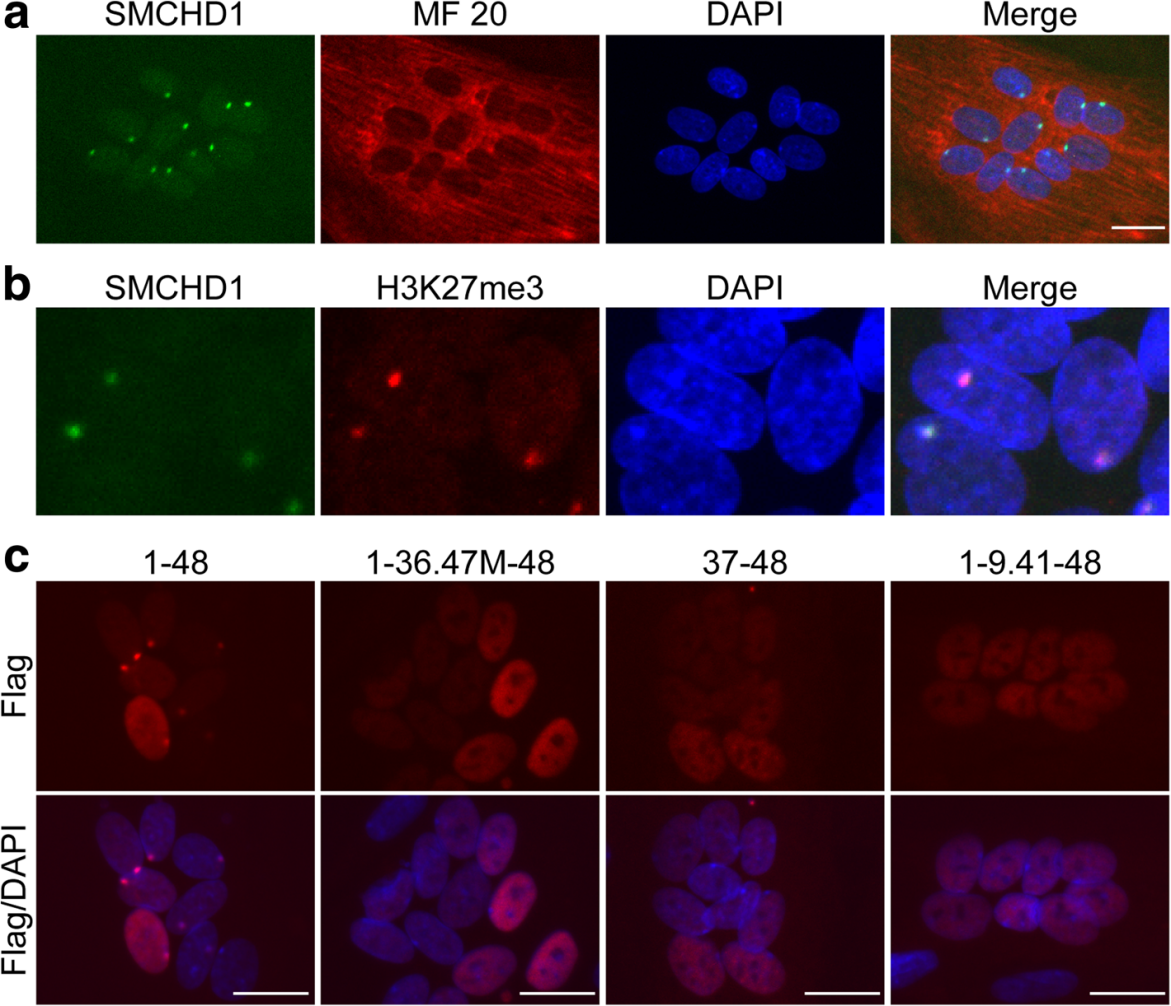
Single spot of SMCHD1 in female myotube. **a** Immunofluorescence for SMCHD1 and myosin heavy chain (MF20 antibody) in control female myotubes. **b** Immunofluorescence for SMCHD1 and H3K27me3 in control female myotubes. **c** Immunofluorescence for Flag in control female myotubes transduced with Flag-tagged SMCHD1 lentivirus. DAPI was used for staining the nucleus. Scale bar is 20 μm. Single spot of full length was confirmed by repeat experiments

In control (MB135) myotubes in differentiation medium, SMCHD1 was mostly localized to one single spot in the nucleus (Fig. 3a). Taking into account that MB135 muscle cells were derived from a female, we tested whether the single spot in myotube nuclei could be co-localized with the inactive X chromosome marker H3K27me3 (histone H3 trimethyl lysine 27) [20, 21] and determined that the spots for SMCHD1 and H3K27me3 were co-localized (Fig. 3b). Similarly, the expression of Exon1-48-Flag also localized to nuclear foci, although the overexpression showed some additional homogeneous nuclear staining. In contrast, all of the mutants Exon1-36.47M-48-Flag, Exon3748-Flag, and Exon1-9.41-48-Flag showed only diffuse nuclear staining with no evident foci (Fig. 3c), suggesting that the structural integrity of SMCHD1, rather than a single specific region, was necessary for the foci and presumably binding to the inactive X chromosome.

### Identification of regions necessary for SMCHD1 homo-dimerization

**Fig. 4:**
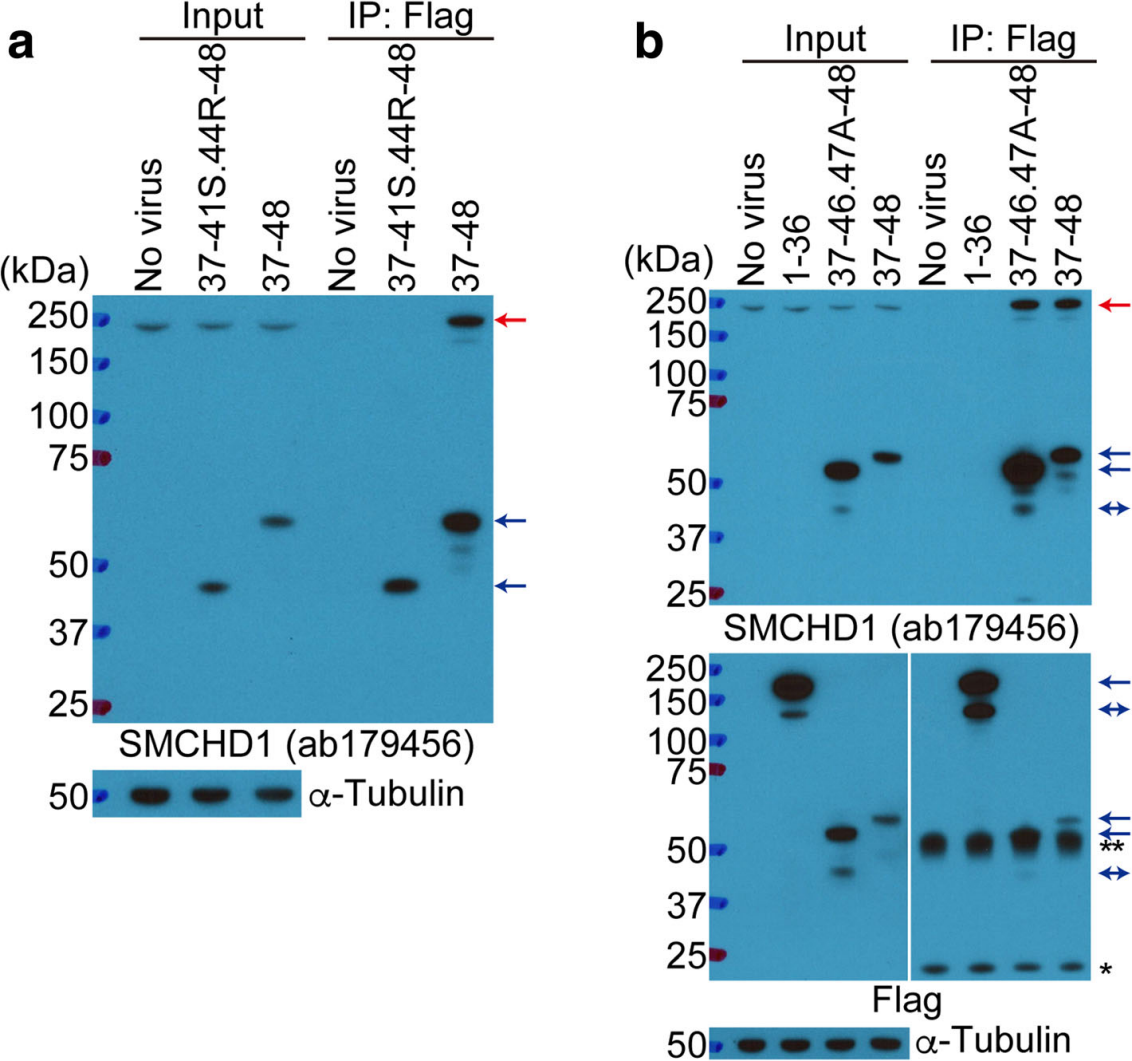
Identification of regions necessary for SMCHD1 homo-dimerization. **a, b** IP of exogenous SMCHD1 in control myoblasts transduced with Flag-tagged SMCHD1 lentivirus followed by Western blot with an anti-SMCHD1 antibody (ab179456) detecting the carboxy-terminal region of SMCHD1. For confirming the expression of Exon1-36-Flag, anti-Flag antibody was used after stripping. α-Tubulin was used as a loading control. Red and blue arrows identify endogenous and exogenous SMCHD1, respectively. Blue double arrows identify smaller fragments of exogenous SMCHD1. IgG heavy chain (**) and light chain (*). IP (immunoprecipitation)

Previous studies using IP showed that the hinge domain was important for SMCHD1 homo-dimerization [8, 10]. We used IP to determine whether different SMCHD1 mutants would dimerize with endogenous SMCHD1. Confirming the prior results, Exon37-48-Flag bound to endogenous SMCHD1, whereas Exon37-41S.44R-48-Flag, which lacks the hinge domain, did not bind to endogenous SMCHD1 (Fig. 4a). Moreover, Exon1-36-Flag, which does not have a hinge domain, also did not bind to endogenous SMCHD1, whereas Exon37-46.47A-48-Flag, which lacks the NLS but has the hinge domain, also bound to endogenous SMCHD1 (Fig. 4b). These results indicate that the hinge domain can mediate dimerization between endogenous SMCHD1 and the mutant SMCHD1 proteins. It is interesting to note that the expression of these mutant SMCHD1 proteins did not alter the abundance of the endogenous SMCHD1 protein, whether the mutant contained the hinge domain or the NLS, or not (Fig. 4a, b), indicating that mutations in SMCHD1 might not alter the abundance of the endogenous SMCHD1 in FSHD2 but might alter its function by forming inactive heterodimers.

**Fig. 5:**
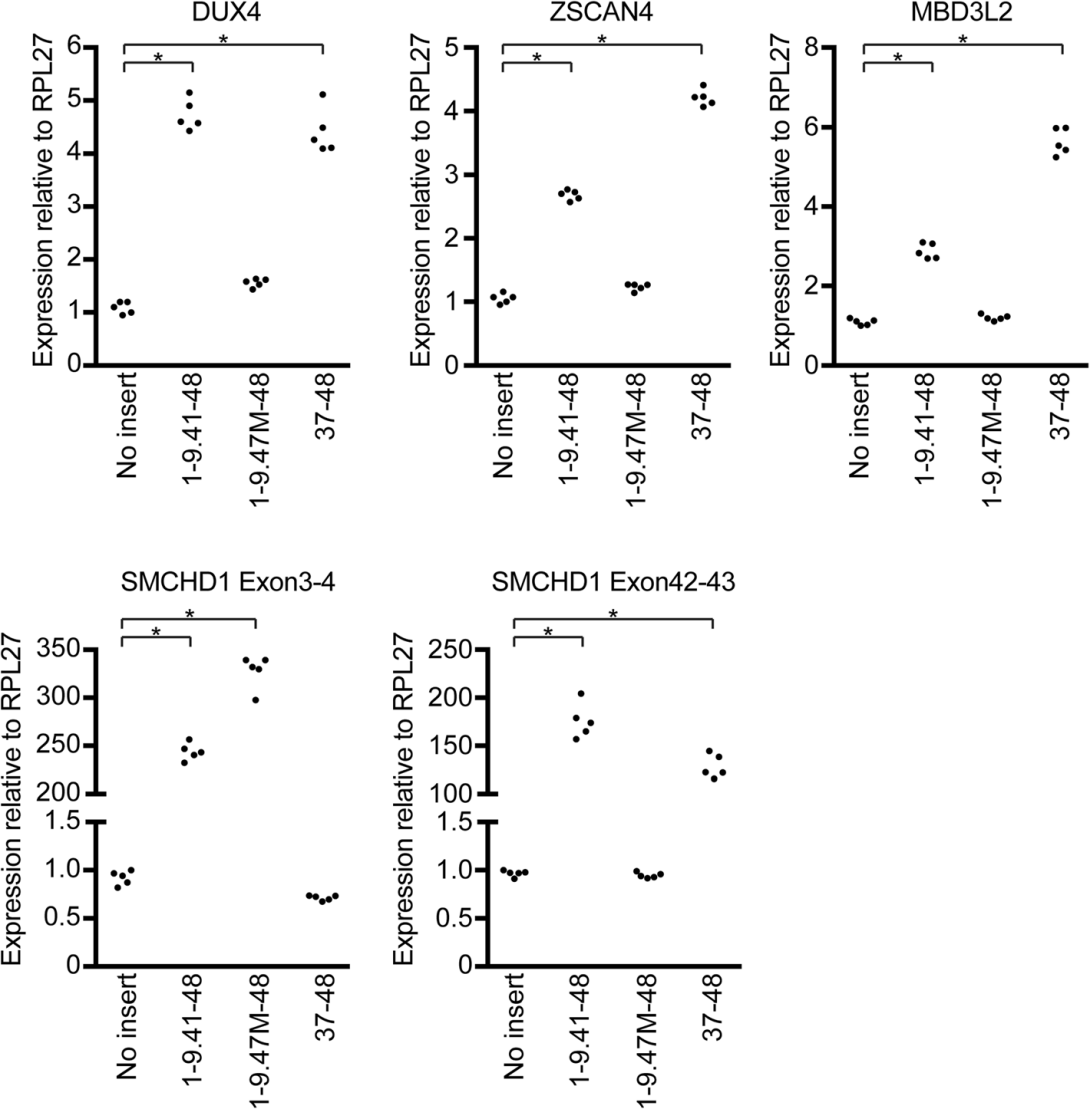
DUX4 expression in FSHD1 myoblasts with mutant SMCHD1. RT-qPCR for DUX4, ZSCAN4, MBD3L2, and SMCHD1 in FSHD1 myoblasts transduced with different mutant SMCHD1 constructs. RPL27 was used as an internal control. (*n* = 5 in each group) Dunnett’ s test (*P < 0.01). These are representative data from three experiments

To determine whether a mutant SMCHD1 capable of forming a heterodimer with the wild-type might partly interfere with normal function, we overexpressed SMCHD1 mutants in FSHD1 myoblasts and measured the level of DUX4 expression. Exon1-9.41-48-Flag and Exon37-48-Flag increased DUX4 and DUX4 target genes (ZSCAN4 and MBD3L2) expression, whereas Exon1-9.47M-48-Flag had less effect on DUX4 and its target genes expression (Fig. 5).

### Identification of cleavage sites in the SMCHD1 protein

**Fig. 6:**
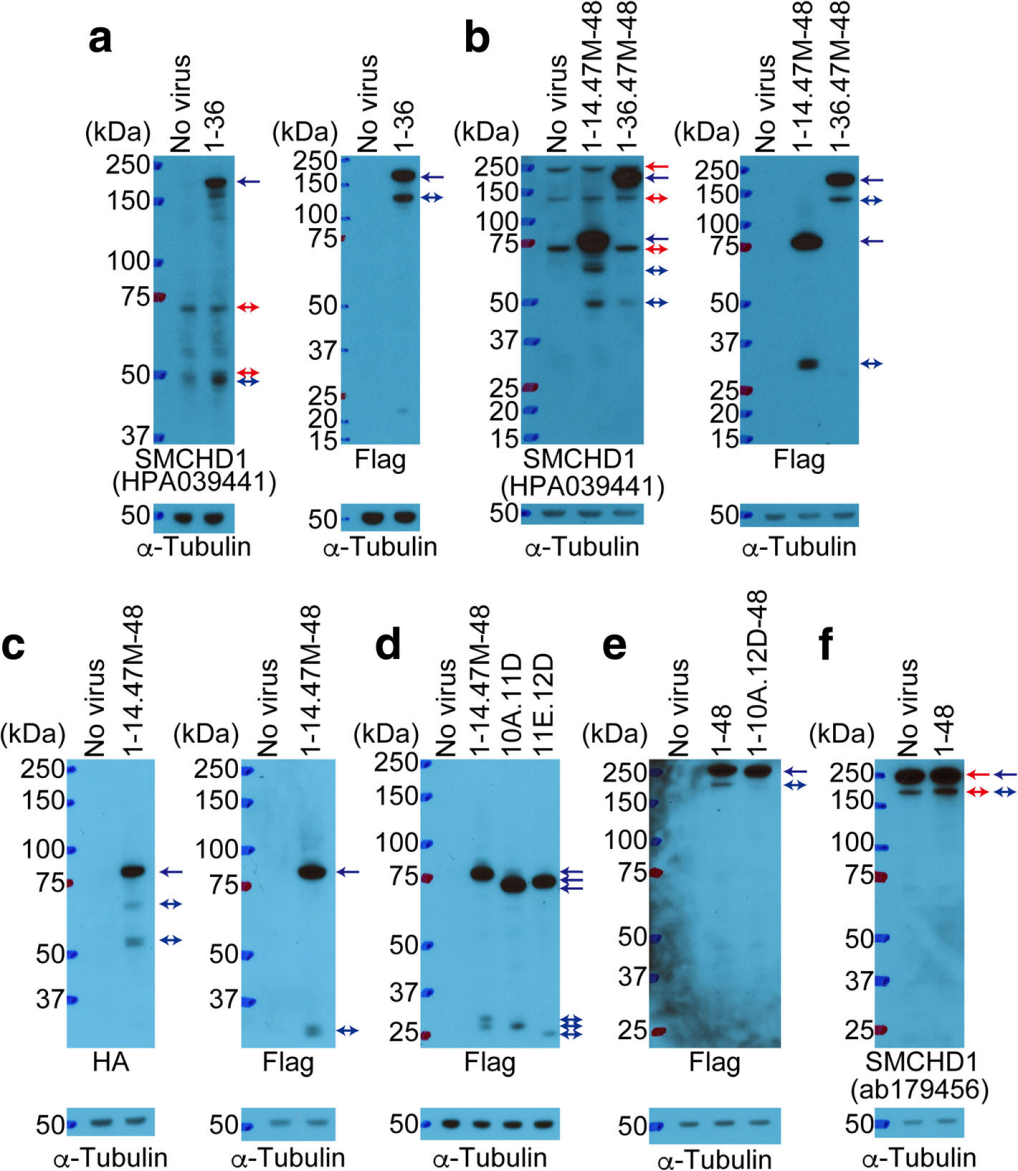
Identification of the cleavage sites. **a**, **b**, and **d–f** Western blot for SMCHD1 (HPA039441 and ab179456) and Flag in control myoblasts transduced with Flag-tagged SMCHD1. (**c**) Western blot for HA and Flag in control myoblasts transduced with HA-Exon1-14.47M-48-Flag. α-Tubulin was used as a loading control. Red and blue arrows indicate endogenous and exogenous SMCHD1, respectively. Red and blue double arrows indicate smaller fragments of endogenous and exogenous SMCHD1, respectively. 10A.11D (Exon1-10A.11D-14.47M-48). 11E.12D (Exon1-11E.12D-14.47M-48). The smaller band from full length was confirmed by repeat experiments

Unexpectedly, in addition to the band of predicted molecular size for the mutant SMCHD1 proteins, we also detected a smaller band(s) (see double arrows in Fig. 4b). To better understand how the smaller bands were produced, we focused on the small band derived from Exon1-36-Flag. We first determined whether the smaller fragment might represent a cleavage product of SMCHD1 comparing immunoreactivity to antibodies directed to either the amino- or carboxy-terminus of the protein. Antibody HPA039441 detected both the predicted full-length protein (173.9 kDa) and a prominent smaller band (about 50 kDa), whereas the anti-Flag antibody detected the predicted full-length protein and a smaller band (about 125 kDa) (Fig. 6a). The total size of the two smaller bands, 50 and 125 kDa, would add together to become a predicted size of the full-length protein. Similar to this, we confirmed the total size of the smaller bands adds to the size of the full-length band in Exon1-36.47M-48-Flag (Fig. 6b).

To narrow down the location of a potential cleavage site, we tested Exon1-14.47M-48-Flag. The HPA039441 detected the predicted band (82.4 kDa) and two smaller bands (approximately 50 and 60 kDa), whereas the anti-Flag antibody detected the full-length band and a smaller band (about 30 kDa) (Fig. 6b). Similarly, the added sizes of the smaller bands (50 and 30 kDa) match the size of the full-length band. Moreover, we tested HA-Exon1-14.47M-48-Flag, which has the HA tag in the amino-terminus and the Flag tag in the carboxy-terminus. Similar to Exon1-14.47M-48-Flag, we detected smaller bands using the anti-HA or Flag antibody (Fig. 6c).

To further narrow down the site(s) of cleavage, we tested Exon1-10A.11D-14.47M-48-Flag (10A.11D), which lacks the 44 aa between 409 and 452 aa in the area of cleavage, and Exon1-11E.12D-14.47M-48-Flag (11E.12D), which lacks the 37 aa between 453 and 489 aa. The anti-Flag antibody detected two smaller bands in Exon1-14.47M-48-Flag, whereas it detected only a single smaller band (about 30 kDa) in 10A.11D and 11E.12D (Fig. 6d). These results indicate that there might be one cleavage site between 409 and 452 aa and a second site between 453 and 489 aa. Next, we tested Exon1-10A.12D-48-Flag, which lack these cleavage sites from full length, and investigated whether there is any difference between Exon1-48-Flag and Exon1-10A.12D-48-Flag. We detected a smaller band from Exon1-48-Flag not Exon1-10A.12D-48-Flag (Fig. 6e). Moreover, antibody ab179456, which recognizes the carboxy-terminus, detected a smaller band in not only Exon1-48-Flag but also endogenous SMCHD1 (Fig. 6f). Together, these results suggest that specific cleavage sites in the ectopic constructs could be also used in the endogenous SMCHD1.

## Discussion

The ATPase domain and the hinge domain make up only 15% of the total protein, and it is important to progressively annotate additional functional domains to help identify functionally significant polymorphisms. In this study, we identified the nuclear localization signal, confirmed the dimerization domain, and identified cleavage sites in the SMCHD1 protein. The NLS of SMCHD1 was mapped from aa 1961 to 1992. The four residues (KRMR) in the 32 amino acids is consistent with the consensus sequence of K(K/R)X(K/R) for a classical NLS [18]. In addition to nuclear localization, the localization of SMCHD1 was observed as a bright intra-nuclear spot that co-localized with a marker for the inactive X chromosome [10, 13–15].

Consistent with previous studies [8, 10], the hinge domain was important for dimerization between endogenous and exogenous SMCHD1. Since a variety of mutations in SMCHD1 coding region have been reported that have the potential to produce different portions of the SMCHD1 protein and might act as either haploinsufficient or possibly dominant negatives [4, 19, 22], understanding the domains that confer functional interactions, such as NLS and homo-dimerization, will be important to elucidate the molecular mechanisms for the mutant SMCHD1 regulation of DUX4 expression. In this regard, it is interesting to note that based on our results with different SMCHD1 proteins, the amount of endogenous SMCHD1 was not altered regardless of the presence or absence of a hinge domain in the mutant; however, it is possible that the dimerization with a mutant partner might alter the function of the wild-type SMCHD1 because overexpression of mutants containing the hinge domain resulted in increased expression of DUX4.

A previous study showed that a mutation affecting the activity of the ATPase domain (E147A) or a deletion of the hinge domain failed to localize to the inactive X chromosome in *Smchd1*^*−/−*^ female mouse embryonic fibroblasts [10]. Together with our finding that deletion of multiple different domains also results in a protein that does not localize to a nuclear focus suggests that lenti-viral expression of SMCHD1 might be an approach to probe the functional significance of polymorphisms of unknown significance in SMCHD1.

As an additional finding, we identified cleavage sites in the SMCHD1 protein between aa 409–452 and aa 453–489. We were unable to determine the protease that cleaves at this site. In addition, since we detected additional smaller fragments from the endogenous SMCHD1 (see Fig. 6a, b), it is possible that SMCHD1 might have additional cleavage sites. Further investigation is necessary to determine whether these cleavage sites could be involved in the biological regulation of SMCHD1 degradation.

## Conclusions

We identified regions of SMCHD1 necessary for nuclear localization, confirmed the region necessary for dimerization, and identified cleavage sites using lenti-viral vectors that express Flag-tagged full-length or different mutant SMCHD1 proteins.

## Additional files

Additional file 1: Sequences of the primers for constructions. (XLSX 23 kb)

Additional file 2: Sequences of the primers for RT-qPCR. (XLSX 46 kb)

## Abbreviations

10A.11D: Exon1-10A.11D-14.47M-48-Flag
11E.12D: Exon1-11E.12D-14.47M-48-Flag
aa: Amino acid
FSHD: Facioscapulohumeral muscular dystrophy
IP: Immunoprecipitation
NLS: Nuclear localization signal
